# Engineering mesophilic GH11 xylanase from *Cellulomonas flavigena* by rational design of N-terminus substitution

**DOI:** 10.3389/fbioe.2022.1044291

**Published:** 2022-11-03

**Authors:** Wenzhuo Tian, Ziyang Zhang, Cuiping Yang, Piwu Li, Jing Xiao, Ruiming Wang, Peng Du, Nan Li, Junqing Wang

**Affiliations:** ^1^ State Key Laboratory of Biobased Material and Green Papermaking (LBMP) (Qilu University of Technology), Jinan, China; ^2^ School of Biological Engineering, Qilu University of Technology, Jinan, China

**Keywords:** N-terminus substitution, thermostability, xylanase, glycoside hydrolase family 11, Cellulomonas flavigena

## Abstract

Xylanase, a glycoside hydrolase, is widely used in the food, papermaking, and textile industries; however, most xylanases are inactive at high temperatures. In this study, a xylanase gene, *CFXyl3*, was cloned from *Cellulomonas flavigena* and expressed in *Escherichia coli* BL21 (DE3). To improve the thermostability of xylanase, four hybrid xylanases with enhanced thermostability (designated EcsXyl1–4) were engineered from CFXyl3, guided by primary and 3D structure analyses. The optimal temperature of CFXyl3 was improved by replacing its N-terminus with the corresponding area of SyXyn11P, a xylanase that belongs to the hyperthermostable GH11 family. The optimal temperatures of the hybrid xylanases EcsXyl1–4 were 60, 60, 65, and 85°C, respectively. The optimal temperature of EcsXyl4 was 30 C higher than that of CFXyl3 (55°C) and its melting temperature was 34.5°C higher than that of CFXyl3. After the hydrolysis of beechwood xylan, the main hydrolysates were xylotetraose, xylotriose, and xylobiose; thus, these hybrid xylanases could be applied to prebiotic xylooligosaccharide manufacturing.

## 1 Introduction

Xylanases are hydrolases that catalyze the breakdown of the β-1,4-glycosidic bonds present in xylan (Scheller et al., 2010). They are the major constituents of hemicelluloses in plant cells and the second most abundant polysaccharides in nature, following cellulose (Thomson, 1993). Thus, xylanases are considered crucial enzymes to exploit lignocellulosic resources (Feng et al., 2015). Since the 1980s, xylanase has been widely applied in the baking and brewing industries, paper pulp and animal feed manufacturing, the treatment of plant cells, and the retting of flax fibers, as well as in the production of biofuels and surfactants (Sharma et al., 2018). In pulping and papermaking, xylanase pretreatment can be used to enhance the brightness of the finished paper (Yang et al., 2019). In addition to removing the hemicellulose from the pulp, xylanase can reduce the amount of chlorine used in bleaching during the entire process (Prabhjot et al., 2016).

For industrial applications, however, xylanases must possess some distinctive characteristics to tolerate extreme conditions, for instance, acidic and/or alkaline environments or high temperatures (Chang et al., 2017). The sources of xylanase are extensive, and include yeast, bacteria, protozoans, marine algae, crustaceans, snails, seeds, insects, etc., but xylanase from filamentous fungi is considered to have great industrial application potential (Polizeli et al., 2005; Wang et al., 2011). Moreover, several thermophilic xylanases from thermophilic bacteria have been investigated; however, their levels, alkali resistance, specific activity, and other enzymatic properties failed to satisfy industrial demands (Zhang et al., 2014; Das et al., 2016). Therefore, it is necessary to discover novel xylanases with excellent temperature properties or modify the primary and/or three-dimensional (3D) structures of existing mesophilic xylanases through protein/genetic engineering. Over 2,200 different glycoside hydrolase (GH) family 11 xylanase structures have been previously reported (http://www.cazy.org/GH11_structure.html). The similarities between these structures include β-jelly-roll topology with conserved catalytic nucleophiles and acid/base glutamate residues (Bueren et al., 2012).

Several studies have explored the influence of sequence and structure on protein stability and tried to engineer improved xylanases for industrial application (Katewadee et al., 2017; Kyoungseon et al., 2021). To our knowledge, the preferred methods of rational design for xylanase are single amino acid substitution, site-directed mutagenesis, and introduction of disulfide. For example, 1) the optimal pH and catalytic efficiency of Xyn30Y5 was shown to increase after 47 mutants were designed and selected (Lai et al., 2020), 2) the heat resistance of xylanase PjxA increased by introducing a disulfide bridge (Cys2–Cys29) at the N-terminal (Xiong et al., 2019), 3) the catalytic performance of xylanase XT6 was improved by single amino acid substitutions (V161L and P209L) (Azouz et al., 2020), and 4) enzyme stability was improved by inducing conformational rigidity by substituting valine and proline at the fifth and sixth residue positions of asparagine (Bhat et al., 2021). Moreover, analysis of the secondary and 3D structure of GH11 xylanase showed that the N-termini have little effect on the stability of the overall structure (Hakulinen et al., 2003; Dumon et al., 2008). The effect of unstructured amino acids in the N-terminal region on xylanase thermostability has also been demonstrated in previous studies (Xue et al., 2012; Lu et al., 2016). Therefore, N-terminal editing using rational design strategies may improve the thermostability of xylanase (Ventorim et al., 2018). In our study, the xylanase CFXyl3, which is stable at pH 8–12 and has a neutral optimal pH (Lisov et al., 2017), was used to explore the alkali and thermal tolerance of xylanase. Hence, the appropriate N-terminus of CFXyl3 was substituted with the corresponding area of SyXyn11P by rational design. Subsequently, four hybrid xylanase genes were constructed and expressed in *Escherichia coli* (DE3), and the enzymatic properties of EcsXyl1–4 were characterized. This genetic engineering strategy is expected to help improve the tolerance characteristics of xylanase, thereby expanding its application scope.

## 2 Materials and methods

### 2.1 Strains, vectors, and culture conditions

The recombinant vectors, pET-28a (+)–CFXyl3 and pET-28a (+)–SyXyn11P, were constructed using the sequences of CFXyl3-and SyXyn11P-encoding genes synthesized by GenScript (Piscataway, NJ, United States). *E. coli* BL21 (DE3) competent cells (Vazyme, Nanjing, China) were used for cloning and expression experiments. Briefly, the bacteria were cultured at 37°C in Luria–Bertani medium containing (w/v) 1% tryptone, 1% NaCl, 0.5% yeast extract, pH 7.0, and 30 μg/ml Kanamycin, as necessary. The following reagents were used: 2 × Phanta Max Master Mix (Dye Plus), ClonExpress Ultra One Step Cloning Kit (C115), Fast Pure Gel DNA Extraction Mini Kit (all from Vazyme), Plasmid Minipreparation Kit (Tiangen Biotech, Beijing, China), and Ni-NTA His Bind Resin (7sea Biotech, Shanghai, China) for protein purification. Beechwood xylan (Harvey Bio, Beijing, China) was used to analyze the enzymatic activity. Unless otherwise specified, all other chemicals used were of analytical grade.

### 2.2 Artificial synthesis of CFXyl3 and SyXyn11P

The amino acid sequences of CFXyl3 (GenBank accession no. WP_013115499.1) and SyXyn11P (GenBank accession no. JX459567) were obtained from the National Center for Biotechnology Information database (http://www.ncbi.nlm.nih.gov/) and their signal peptides were predicted using SignalP (http://www.cbs.dtu.dk/services/SignalP/). Both CFXyl3 and SyXyn11P, to which *Nco*I (CCATGG) and *Xho*I (CTCGAG) restriction sites were fused at their 5′- and 3′-ends, were inserted into the pET-28a (+) vector. CFXyl3 and SyXyn11P showed bias toward *E. coli* BL21 (DE3) by optimizing synonymous codons.

### 2.3 Analysis of the primary and 3D structures

Sequence homology was evaluated using BLAST (https://blast.ncbi.nlm.nih.gov/Blast.cgi). Homology modeling was accomplished using SWISS–MODEL (https://swissmodel.expasy.org/). Multiple alignment of the primary structures was achieved using ESPRIPT v3.0 (http://www.ebi.ac.uk/ESPRIPT), ClustalW (http://www.ebi.ac.uk/ClustalW), and DNAMAN v9.0 (https://www.lynnon.com) software. 3D structure analysis was performed using PyMOL 2.5 software (Schrödinger, New York, NY, United States).

### 2.4 Construction of hybrid xylanase gene

Hybrid xylanase genes (*EcsXyl1*, EcsXyl*2*, *EcsXyl3*, and *EcsXyl4*), were constructed by replacing the 5′-end nucleotide sequence (106, 117, 132, and 144 bp, respectively) of *CFXyl3* with the corresponding segment (115, 126, 141, and 153 bp, respectively) of *SyXyn11P* by seamless cloning. All primers designed for plasmid construction are listed in Supplementary Table S1. The plasmid pET-28a (+)–CFXyl3 was used as a template with the primers CFXyl3-1, CFXyl3-2, CFXyl3-3, and CFXyl3-4, and the plasmid pET-28a (+)–SyXyn11P was used as a template with the primers SyXyn11P-1, SyXyn11P-2, SyXyn11P-3, and SyXyn11P-4. The PCR reaction was operated as formerly described (Gao et al., 2013). The PCR products were gel-purified and digested with the *Dpn*1 restriction enzyme, and the four pairs of PCR products were then linked using the ClonExpress Ultra One Step Cloning Kit, resulting in the recombinant vectors pET-28a (+)–EcsXyl1, pET-28a (+)–EcsXyl2, pET-28a (+)–EcsXyl3, and pET-28a (+)–EcsXyl4, which were then integrated into the *E. coli* BL21 (DE3) genome for amplification and preparation. The original plasmid pET-28a (+)–CFXyl3 was also transformed into *E. coli* BL21 (DE3) as a control. The integration of CFXyl3 and SyXyn11P into the genome of *E. coli* BL21 (DE3) was confirmed by genome sequencing (Sangon Biotech, Shanghai, China).

### 2.5 Expression and purification of the hybrid xylanases

To efficiently express EcsXyl1–EcsXyl4, seed cultures (5 ml) of *E. coli* BL21 (DE3) harboring the recombinant plasmids were created by culturing the cells on a shaker at a rotation speed of 200 r/min for approximately 12 h at 37°C. The seed culture was further expanded in Luria-Bertani medium (50 ml, pH 7.0) containing kanamycin (30 μg/ml) at 37°C on a rotary shaker (200 r/min). IPTG was added to a final concentration of 1 mM when the optimal density of the cultured broth at 600 nm was 0.6–0.8. Incubation was continued for 20–22 h at 25°C. Next, the induced *E. coli* cells were collected from 20 ml of cultured broth by high-speed centrifugation, resuspended, and washed twice in the same volume of 20 mm Gly–NaOH buffer (pH 7.0). After ultrasonication for 10 min, the supernatant was concentrated to 1 ml by high-speed centrifugation and purified by Ni–NTA His Bind Resin, followed by elution with different concentrations of imidazole buffer at a natural flow rate. Aliquots of 1.5 ml of purified EcsXyl1, EcsXyl2, EcsXyl3, and EcsXyl4 enzyme solution were collected for further determination. Unless otherwise specified, the purification step was performed at 4°C. The molecular weight of the pure proteins was determined by sodium dodecyl sulfate-polyacrylamide gel electrophoresis using 10% homogeneous polyacrylamide gel (Djekrif et al., 2021). The concentration (mg/ml) of protein was determined using a BCA Protein Assay Kit.

### 2.6 Characterization of the hybrid xylanases

The activity of the hybrid xylanases was determined by quantifying the amount of released reducing sugars from beechwood xylan using the 3,5-dinitrosalicylic acid (DNS) method and xylose as the standard (Miller et al., 1960). Reaction mixtures containing 500 μl of enzyme (diluted with Gly–NaOH buffer, pH 7.0) and 500 μl of the 1.0% (w/v) beechwood xylan solution (diluted with Gly–NaOH buffer, pH 7.0) were reacted at 55°C for 15 min. Then, 1.25 ml of the DNS reagent was added to the samples in a boiling water bath for 5 min to finish the reaction, and the amount of released reducing sugars was determined by measuring the absorbance of the samples at 540 nm (Moukouli et al., 2011). One unit of xylanase activity was defined as the amount of enzyme that formed reducing groups corresponding to 1 μmol of xylose in 1 min under the assay conditions (at pH 7.0 and 55°C for 15 min). All enzyme activity measurements were performed in triplicate and the average value of three experiments was reported.

#### 2.6.1 Optimal temperature and thermostability of the hybrid xylanases

The optimal temperature of the hybrid xylanases was evaluated between 55 and 90°C (intervals: 5°C) at pH 7.0 for 15 min. To investigate their thermal stability, CFxyl3 and EcsXyl1-4 were tested in the absence of beechwood xylan at various temperatures (50, 60, and 70°C) for 30–150 min. The thermostability of xylanase before incubation was defined as 100%.

#### 2.6.2 Optimal pH and alkali stability of the hybrid xylanases

The optimal pH of the hybrid xylanases was determined under the standard assay conditions and 1.0% (w/v) of beechwood xylan dissolved in various buffers over a pH range of 3.0–12.0. To estimate their pH stability, xylanases were incubated at 25°C for 16 h (Tang et al., 2021) at varying pH values (Na_2_HPO_4_–citric acid buffer: pH 3.0–7.0; Glycine-NaOH buffer: pH 8.0–12.0) before their enzyme activities were determined. The pH stability of xylanase at the initial activity level determined before incubation was defined as 100%.

#### 2.6.3 Effect of metal ions

To assess the effect of metal ions on the enzymatic activity, aliquots of the xylanase and substrate were reacted at 55°C with a series of salts for 15 min. The following metals were used: KCl, CaCl_2_, NaCl, MgCl_2_, ZnCl_2_, CuSO_4_, BaCl_2_, MnSO_4_, FeSO_4_, FeCl_3_, NiCl_2_, and CoCl_2_. The final concentration of these salts in the entire reaction system was 1 mm. Xylanase solution without any added compounds was used as the control (defined as 100%).

### 2.7 Measurement of the melting temperature

The T_m_ is defined as the temperature at which half of a protein’s 3D structure is unfolded. The higher the T_m_ value of a protein or enzyme, the more thermostable its 3D structure (Zhang et al., 2014). The T_m_ value of xylanase was determined using a Protein Thermal Shift (PTS) Kit and regarded as the temperature corresponding to the peak value in the derivative melting curve.

### 2.8 Determination of hydrolysis products

Beechwood xylan suspension (1 g/L) prepared in Gly–NaOH buffer (pH 7.0) was reacted with EcsXyl1–EcsXyl4 at 55°C for 15 min. The samples were placed in a boiling water bath for 5 min to finish the hydrolytic reaction. The standard xylose (X1), xylobiose (X2), xylotriose (X3), xylotetraose (X4), xylopentaose (X5), and xylohexaose (X6) (Solarbio, Beijing, China) and hydrolysates released from beechwood xylan were evaluated by high-performance liquid chromatography. Isolation of the produced sugars was performed using a Hi-Plex Ca column (300 mm × 7.7 mm; Agilent, Santa Clara, CA, United States); the mobile phase was pure water and the flow rate was 0.6 ml/min. The column temperature was maintained at 80°C and 10 μL of the sample was injected. Sugar peak areas were detected using a Shimadzu RID–10A refractive index detector. The peak time was compared between each hydrolysate and the corresponding standard to determine the type of hydrolysate.

### 2.9 Molecular dynamic simulations and protein interaction analysis

The X-ray crystal structure of SyXyn11P was obtained from the Protein Data Bank (PDB, https://www.rcsb.org). 3D models of CFXyl3 and the four hybrid xylanases were built using the SWISS–MODEL server (https://swissmodel.expasy.org) with SyXyn11P (2VUL) as the template. MD simulations were accomplished using GROMACS v4.5.4 (https://www.gromacs.org) with the following settings: a GROMOS96 43 aL force field and the SPC/E extended simple point charge as the water model. All crystalline molecules unrelated to the protein structure (including water) were removed and the protein was dissolved in a cubic box, in which the closest distance between the periodic box and protein atom was set as 15 Å. Chloride ions and sodium were added to neutralize the systems. The system was energy-minimized following steepest descent methods (maximum steps of 2,000) and the conjugate gradient method with Cα restrained before the simulation. The energy minimization was then iterated without atom restraint. The simulation for production running was performed for 10 ns, with the time step set to 2 fs and temperatures of 300, 350, 400, 450, and 500 K. The RMSD and RMSF were used to analyze protein stability. Finally, the protein interactions of all these structures were determined using the protein interactions calculator (PIC) (http://pic.mbu.iisc.ernet.in/).

### 2.10 Enzymatic kinetic parameters

The hydrolysis reaction rates (mmol/min/mg) of CFXyl3 and EcsXyl4 were determined at 55°C and pH 7.0 for 15 min at beech xylan concentrations of 1.0–10.0 mg/mL. A plot of reaction rate versus substrate concentration was plotted to verify that the hydrolysis modes of CFXyl3 and EcsXyl4 conform to the Michaelis–Menten equation. The kinetic parameters, K_m_ and V_max_ values, were graphically determined using a nonlinear curve fit.

## 3 Results and discussion

### 3.1 Analysis of the primary and 3D structures

**FIGURE 1:**
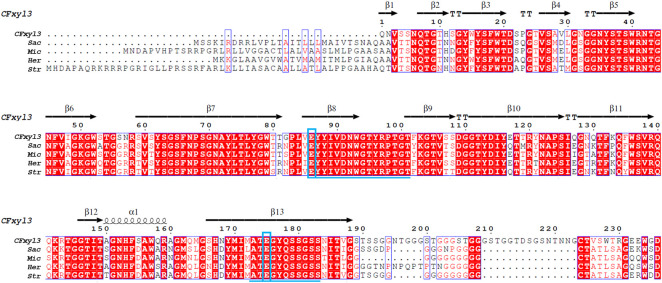
Primary structures multiple alignment between CFXyl3 and four typical xylanases from family 11. Abbreviations: CFXyl3 (WP_013115499.1, in this work); *Str, Streptomyces* (WP_093661817.1); *Sac, Saccharothrix* (NUT48201.1); *Mic, Micromonospora* (WP_168002960.1); and *Her, Herbidospora cretacea* (WP_051760875.1). The duplicate residues among these xylanases showed in red background. The two blue boxed letters mark the catalytic residues Glu86 (acid/base) and Glu175 (nucleophile), numbered by CFXyl3. The underlined amino acid sequence indicated two conserved motifs. The catalytic residues are indicated with blue boxes. One α-helix and 13 β-strands in CFXyl3 are represented as a black coil and black arrows, respectively.

Alignment of the amino acid sequence of CFXyl3 from *Cellulomonas flavigena* along those of GH11 xylanases from *Streptomyces* (WP_093661817.1), *Saccharothrix* (NUT48201.1), *Micromonospora* (WP_168002960.1), and *Herbidospora cretacea* (WP_051760875.1) revealed that it had a similarity of 63.36%, 60.69%, 64.68%, and 60.94%, respectively. Two conserved motifs-EYYIVDNWGTYRPTGT and ATEGYQSSGSS—were identified in family 11 xylanases (Figure 1). Furthermore, catalytic residues that are rigidly conserved among family 11 members, acid/base Glu86 and nucleophile Glu175 (numbered by CFXyl3), were found in these enzymes (Figure 1). All the characteristics in Figure 1 indicate that CFXyl3 belongs to GH family 11. Notably, the N-terminal of each sequence was found to vary greatly, which may be a factor affecting the thermostability of the xylanase. Previous studies demonstrated that the introduction of disulfide bridges at the N-terminal could stabilize even thermostable family GH11 xylanases (Wang et al., 2012). Moreover, the conformational rigidity of family GH11 xylanases can be improved by the substitution of the initial amino acid residues of the N-terminal region (Bhat et al., 2021). Hence, it becomes clear that the N-terminal end is critical for the overall stability of the GH family 11 xylanase molecule.

### 3.2 Construction of a hybrid xylanase gene

**FIGURE 2:**
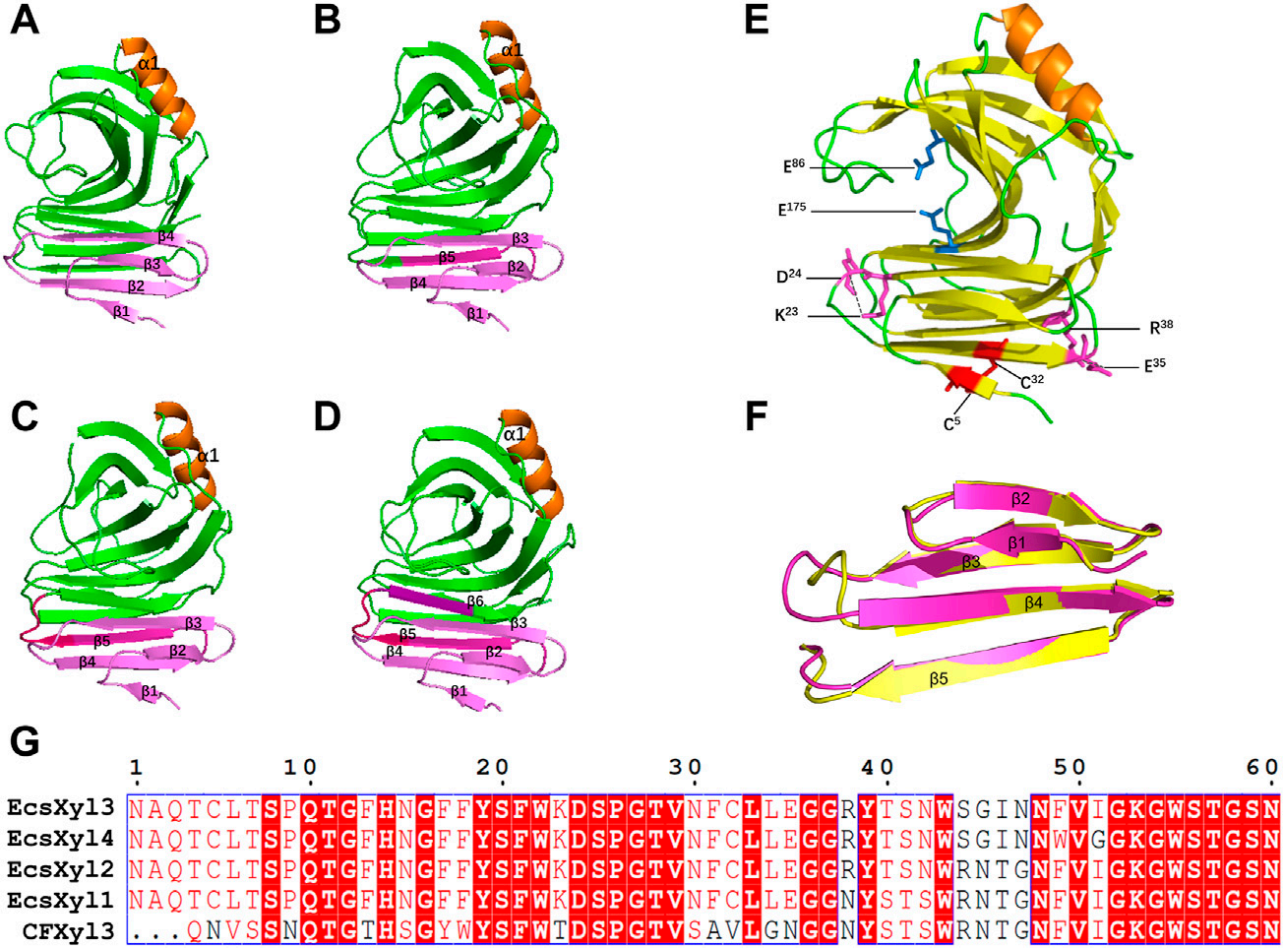
The 3D structure analysis of EcsXyl1 **(A)**, EcsXyl2 **(B)**, EcsXyl3 **(C)** and EcsXyl4 **(D)**. The pink part and purple part are the amino acid position of the N-terminal substitution. **(E)**: The catalytic residues (Glu86 and Glu175) exist in the central part of the active area. The disulfide bridge (Cys5–Cys32) and salt bridges (Lys23–Asp24 and Glu35–Arg38) were led into CFXyl3 arose from N-terminus substitution. **(F)**: Stereoview of superimposed structure of the N-terminus substitution part of the CFxyl3 (yellow) and Ecxyl4 (pink). **(G)**: Sequence alignment of the N-terminus substitution part of the CFXyl3 and EcsXyl1–EcsXyl4.

Based on 3D structure analysis, several β-pleated sheets were identified at the N-terminal region of CFXyl3. Thus, we replaced the first four, five, and six β-pleated sheets (Figures 2A–D) to design novel hybrid xylanases based on CFXyl3 and SyXyn11P, which shared 64.9% of their amino acid sequences. In the first round, SyXyn11P was used as the template to amplify 115, 126, 141, and 153 bp fragments of the *sy* sequence (Figure 2G). Next, CFXyl3 was used as the template for second-round amplification. As a result, four specific fragments of the CFXyl3 (approximately 800 bp each) were amplified and inserted into the pET-28a (+) vector. Finally, the four SyXyn11P fragments were cloned into the four pET-28a (+)–CFXyl3 sequences, respectively, by seamless cloning. Sequencing results certified that the cloned EcsXyl1, EcsXyl2, EcsXyl3, and EcsXyl4 were exactly 927 bp in length, encoded for 309 amino acids, and had predicted isoelectric points of 9.15, 9.31, 9.15, and 9.15, respectively.

### 3.3 Characterization of the hybrid xylanases

**FIGURE 3:**
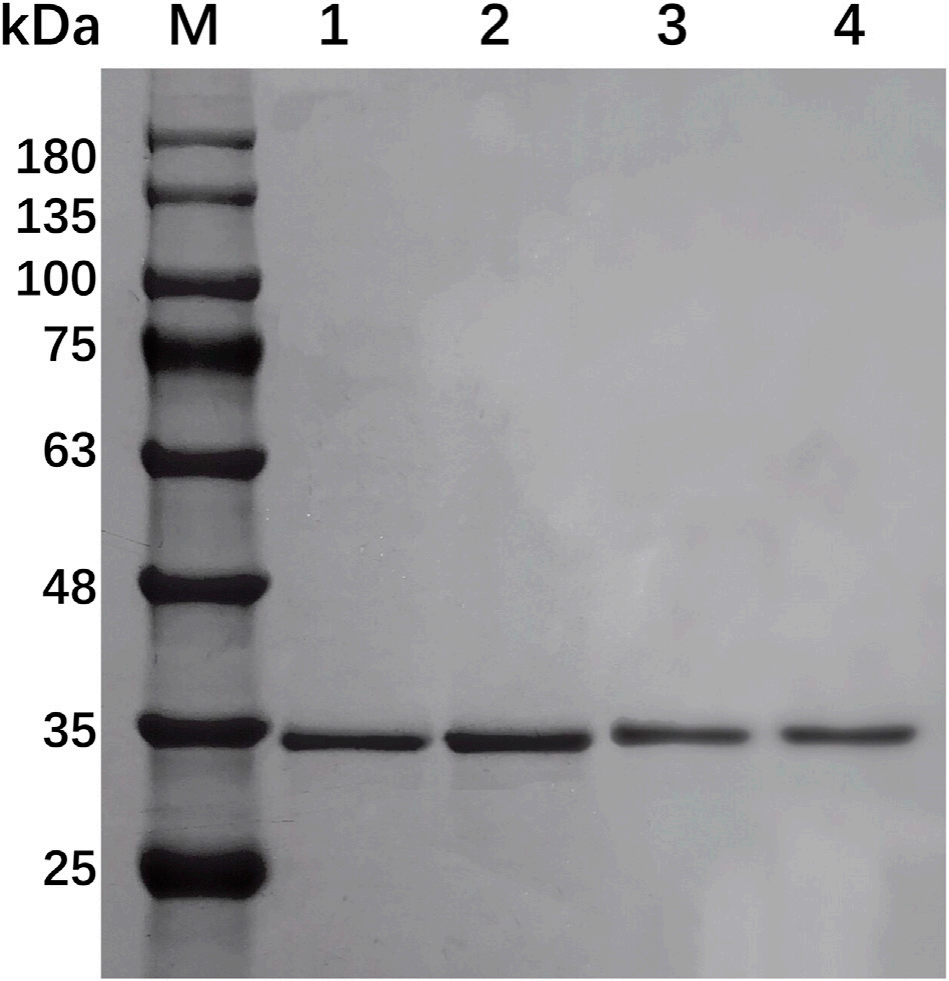
SDS-PAGE of EcsXyl1– EcsXyl4 purification steps. Lane M, molecular weight marker; Lane 1, purified EcsXyl1, Lane 2, purified EcsXyl2, Lane 3, purified EcsXyl3, Lane 4, purified EcsXyl4.

EcsXyl1, EcsXyl2, EcsXyl3, and EcsXyl4 were purified to homogeneity by ultrasonic crushing, high-speed centrifugation, and Ni–NTA His Bind Resin filtration. This led to a final hybrid protein purity of more than 80%, analyzed using SDS-PAGE imaging (Figure 3) and Image Lab 4.1 software. The molecular weight of GH 11 xylanase is usually in the range of 20–30 kDa (Alokika and Singh, 2019). The hybrid xylanases EcsXyl1-4 had a molecular weight of approximately 34.5 kDa, which is higher than most of GH family 11 xylanases from *Chaetomium* sp. (20.6 kDa; Liu et al., 2021), *Streptomyces* sp. strain J103 (24.47 kDa; Marasinghe et al., 2021), and *Myceliophthora heterothallica* F.2.1.4. (24.7 kDa) (de Amo et al., 2019), and lower than that of the xylanase from *Bacillus* sp. 41M–1 (36 kDa) (Takita et al., 2021). The specific activities of the purified EcsXyl1-4 toward beechwood xylan under standard assay conditions were 16.18, 14.44, 29.09, and 80.90 U/mg, respectively. The specific activity of EcsXyl4 increased by 1.54 U/mg, compared with that of CFXyl3 (79.36 U/mg), which may be explained by a significant increase in its optimal temperature (Arcus et al., 2020).

**FIGURE 4:**
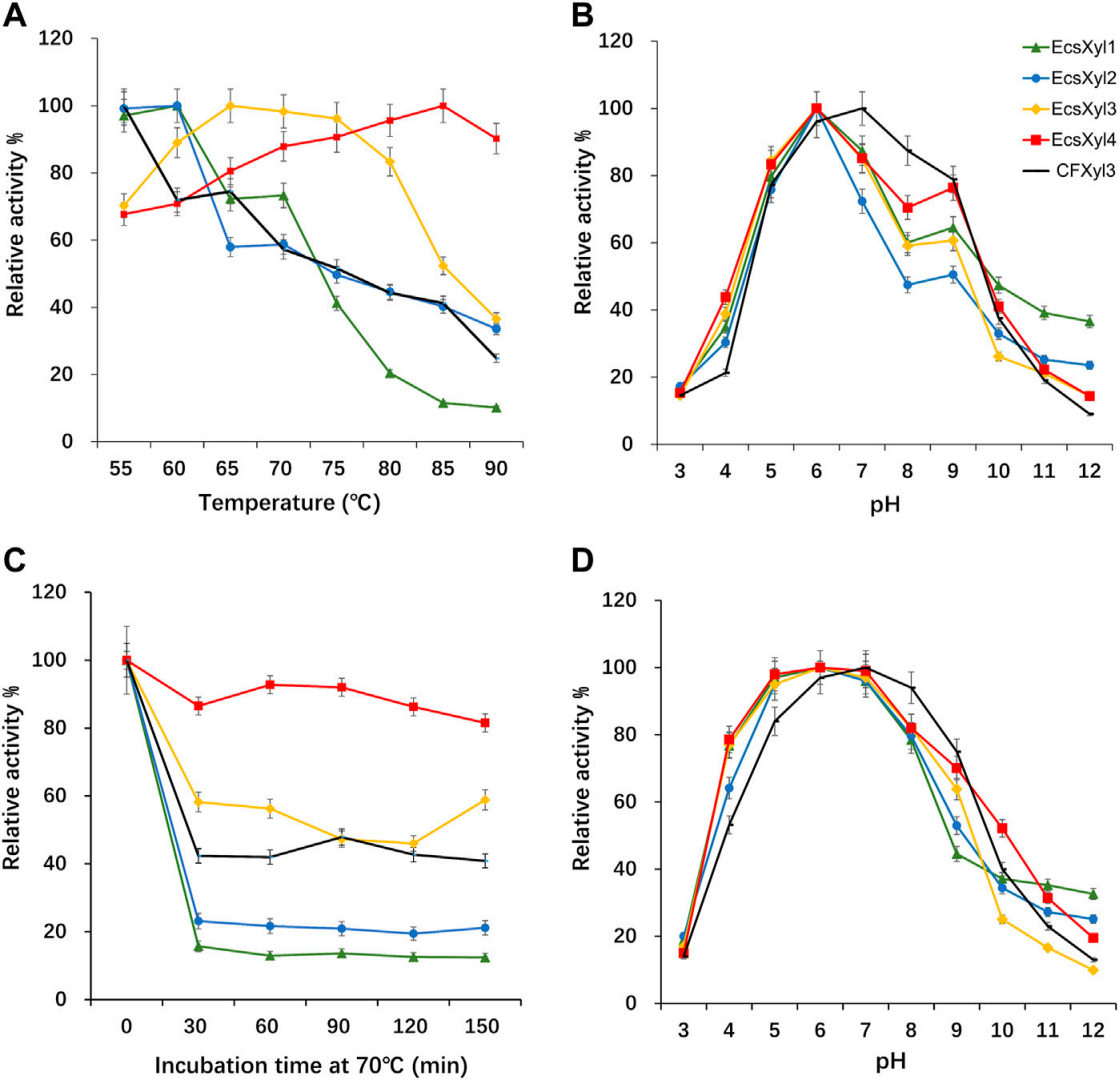
Influence of the temperature and pH on CFXyl3 and EcsXyl1–4 xylanases, thermal and pH stabilities. **(A)**: Optimal temperature; **(B)**: Optimal pH; **(C)**: Thermal stability at 70°C; **(D)**: pH stability.

Analysis of the hybrid enzymes at diverse temperatures (55–90°C) for 15 min revealed that the optimal temperatures for beechwood xylan hydrolysis by EcsXyl1–4 were 60, 60, 65, and 85°C, respectively (Figure 4A). The optimal hydrolysis temperature of CFxyl3 was 55°C. The apparent temperature optimum of EcsXyl4 shifted upwards by 30°C–85 C, which is higher than those of most GH11 family xylanases. Table 1 compared the property of other reported xylanases. The optimum temperature of GH11 xylanase TLX from *Thermomyces lanuginosus* shifted upwards by 10°C–75°C after a disulfide bridge was introduced into the N-terminal region (Wang et al., 2012). The optimum temperature of GH11 xylanase XynJ from *Bacillus* sp. strain 41M–1 shifted upwards by 5 C–65°C owing to random mutations (Takita et al., 2021) and that of Xyn2 from *Trichoderma reesei* shifted upwards by 10°C–60°C after site-directed mutagenesis (He et al., 2019). After incubating the CFXyl3 and four hybrid xylanases at pH 7.0 and 70°C for 30–150 min, the stability of the five xylanases was determined (Figure 4C). EcsXyl4 displayed >80% of its residual activity within 150 min, while CFXyl3 retained about 40% of its residual activity from 30 min. In another study, r-ec-XylMh from *M. heterothallica* maintained a residual activity of approximately 30% of its normal activity when incubated at 70°C for 60 min (de Amo et al., 2019). Additionally, Xyn1923 from *Microbacterium imperiale* YD-01 only maintain 63% of its maximum activity after incubation at 65°C for 30 min, whereas after incubation at 70–80°C for 30 min, no activity was detectable (Tang et al., 2021). Moreover, CFXyl3 and EcsXyl1-3 exhibited lower thermostability than EcsXyl4 at 50, 60, and 70°C (Figure 4C, Supplementary Figure S2). In general, EcsXyl4 showed the best thermostability among the four hybrid xylanases engineered in this study.

**TABLE 1:** Comparison of properties of other reported xylanases.

Xylanase	Optimal pH	Optimal temperature (°C)	Host	Source of microorganism	References
PTxA-DB	3.5	65	*E.coli* BL21	*Penicillium janthinellum*	Xiong et al. (2019)
rXynS1	5.0	55	*E.coli* BL21	*Streptomyces* sp	Marasinghe et al. (2021)
XM1	6.0	60	*Pichia pastoris*	*Trichoderma reesei*	He et al. (2019)
XynA	6.2	65	*E.coli* BL21	*Thermomyces lanuginosus*	Qu et al. (2015)
XynR8	6.5	55	*E.coli* BL21	*Neocallimastigales rumen fungal*	Xue et al. (2012)
XynA	6.5	60	*E.coli* BL21	*Orpinomyces* sp	Ventorim et al. (2018)
CsXyn11A	7.0	70	*Aspergillus niger*	*Chaetomium* sp	Liu et al. (2022)
Xyn11A-LC	7.5	55	*E.coli* BL21	*Bacillus* sp	Bai et al. (2016)
EcsXyl1	6.0	60	*E.coli* BL21	*Cellulomonas flavigena*	This work
EcsXyl2	6.0	60	*E.coli* BL21	*Cellulomonas flavigena*	This work
EcsXyl3	6.0	65	*E.coli* BL21	*Cellulomonas flavigena*	This work
EcsXyl4	6.0	85	*E.coli* BL21	*Cellulomonas flavigena*	This work

After incubation at pH 3.0–12.0 and 55 C for 15 min, the optimal pH values of CFXyl3 and EcsXyl1-4 were determined (Figure 4B). The apparent optimal pH of CFXyl3 was 7.0, whereas that of the four hybrid xylanases was 6.0. This is same as those of the GH family 11 xylanases from *M. heterothallica* (pH 6.0) (de Amo et al., 2019) and *T. reesei* (pH 6.0) (He et al., 2019). It is higher than those of GH family 11 xylanases from *Penicillium janthinellum* MA21601 (pH 4.0) (Xiong et al., 2019), *Streptomyces* sp. J103 (pH 5.0) (Marasinghe et al., 2021), and *Fusarium* sp. 21 (pH 5.0) (Li et al., 2020), but lower than that of a GH family 11 xylanase from *Clavispora lusitaniae* ABS7 (pH 9.0) (Djekrif et al., 2021). To determine pH stability, the xylanases were incubated in buffers with different pH values at 25°C for 16 h, and then the residual activities were measured at 55°C. The pH stability analysis showed that the CFXyl3 at pH 5.0–9.0 retained more than 80% of its maximum activity after incubation, whereas the four hybrid xylanases, with their maximum activities at pH 5.0–8.0, retained more than 78% of their activity (Figure 4D). As the pH increased above 8.0, the hybrid xylanase activity showed a significant downward trend. Figure 4D showed that CFXyl3 and the four hybrid xylanases were unstable under strong acid/alkaline conditions. In another study, a GH family 11 xylanase Xyn1923 from *M. imperiale* YD–01 retained more than 89% and 91% of its enzyme activities at pH 6.0 and 7.0 after incubation at 25 C for 16 h (Tang et al., 2021). Additionally, rXynS1 from *Streptomyces* sp. J103 had a relative activity of over 58% at pH 6.0–7.0 (Marasinghe et al., 2021).

**FIGURE 5:**
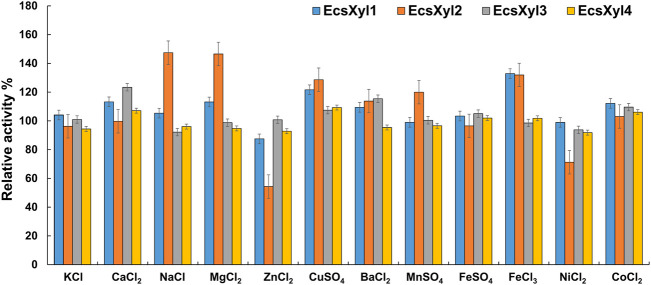
Effect of metal ions on xylanase activity. The activity without any addition was regarded as control. The bars show the hybrid xylanase activity of EcsXyl1– EcsXyl4, respectively, in the presence of metal ions (1 mm).

Figure 5 shows the influence of metal ions on the enzymatic activity of EcsXyl1–4, for EcsXyl1, K^+^, Na^+^, Mn^2+^, Fe^2+^, and Ni^2+^ hardly affected the enzymatic activities, Ca^2+^, Mg^2+^, Cu^2+^, Fe^3+^, and Co^2+^ enhanced its activity, whereas Zn^2+^ significantly inhibited the activity of 87.49%. Similarly, xylanase (BX) from *Clostridium* sp. BOH3 also showed enhanced activity after the addition of 1 mm Ca^2+^, Mg^2+^, Cu^2+^, Fe^3+^, and Co^2+^ (Rajagopalan et al., 2021). In the case of EcsXyl2, Zn^2+^, and Ni^2+^ significantly inhibited activity; however, Na^+^ and Mg^2+^ enhanced activity by 147 and 146%. For EcsXyl3, Ba^2+^ Co^2+^ enhanced its activity and the enzymatic activity of EcsXyl4 was hardly affected by any of the metal ions. Similarly, Na^+^, Mg^2+^, and Ca^2+^ enhanced the activity of xylanase from *Bacillus pumilus* GESF1, while Zn^2+^ strongly inhibited enzyme activity (Menon et al., 2010). Additionally, xylanase EX624 from *Streptomyces* sp. CS624 also displayed enhanced activity in the presence of Ca^2+^ (Mander et al., 2014).

### 3.4 Measurement of the melting temperature

**FIGURE 6:**
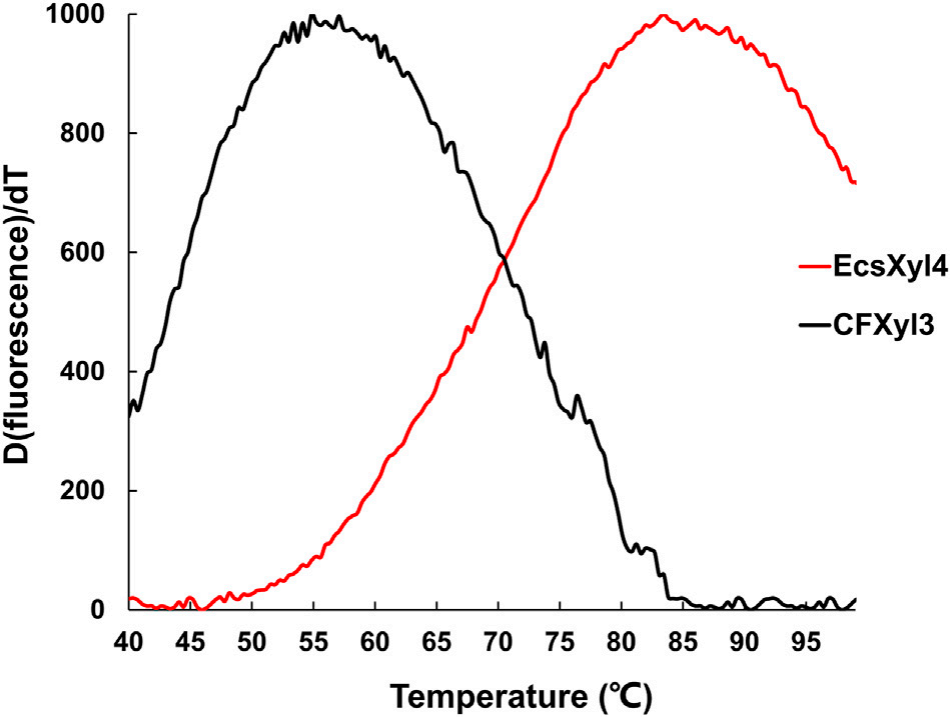
Derivative melting curves of CFXyl3 (*black*) and EcsXyl4 (*red*). The emission intensity of the fluorescence dye was recorded from 40 to 99°C at an elevated rate of 1°C min^−1^.

The emission intensity of the fluorescent dye bound to the hydrophobic region of the protein gradually increases as a protein unfolds at high temperatures (Zhang et al., 2014). Based on this principle, the T_m_ values of CFXyl3 and EcsXyl4 measured from the derivative melting curve were 55.2 and 84.7°C, respectively (Figure 6). Thus, the T_m_ value of EcsXyl4 increased by 34.5°C compared with that of CFXyl3 after N-terminal substitution.

### 3.5 Hydrolysates from beechwood xylan

**FIGURE 7:**
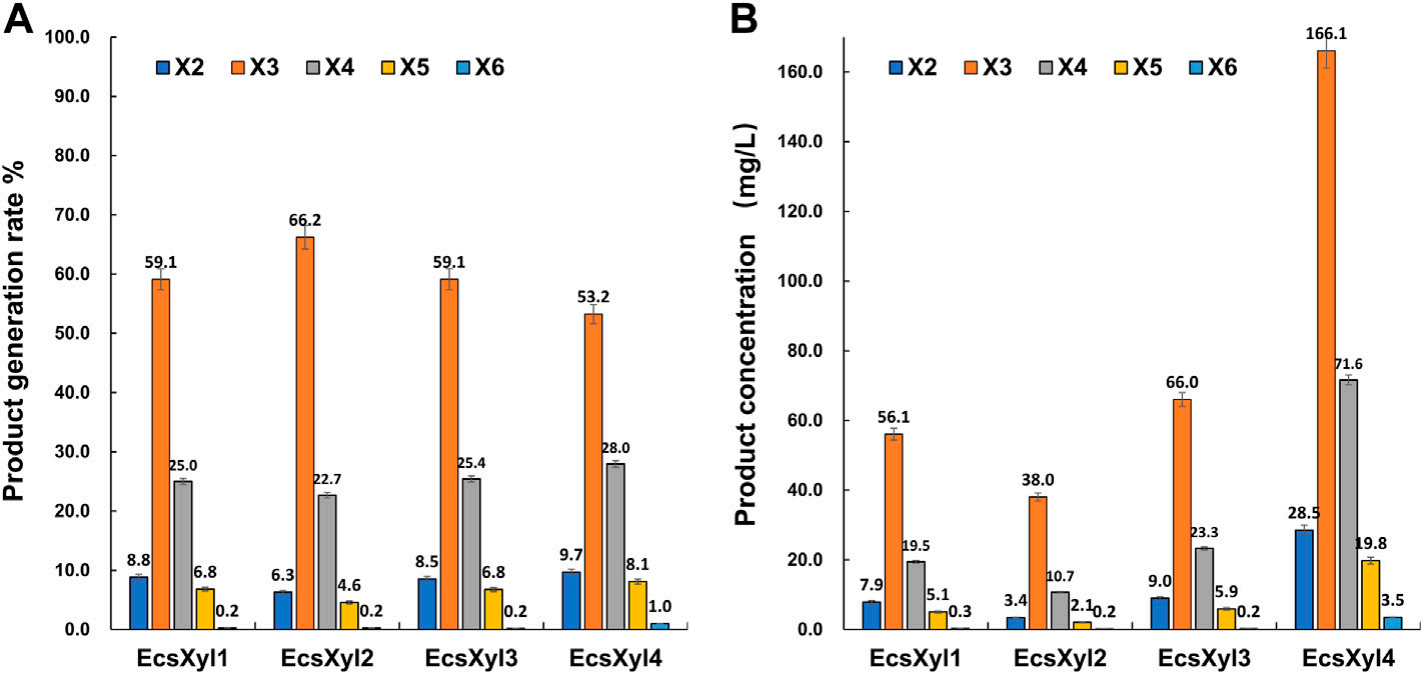
HPLC result analysis of the hydrolystaes broken down into beechwood xylan by EcsXyl1– EcsXyl4 at pH 9.0 and 55°C for 30 min. **(A)**: Product generation rate; **(B)**: Product concentration.

After a reaction at 55 C and pH 7.0 for 30 min, X3 and X4 were detected as the main hydrolysates obtained from insoluble beechwood xylan (1 mg/ml), analyzed using HPLC, whereas xylose was barely detectable (Supplementary Figure S1). As shown in Figure 7, no xylose was detected throughout the hydrolysis of beechwood xylan by the hybrid xylanases, suggesting that EcsXyl1–EcsXyl4 were endo-acting xylanases (Dudkin et al., 1980). Similarly, the main hydrolysates obtained from oat-spelled xylan are X2, X3, and X4, while no xylose was determined after hydrolysis by a xylanase XynA from *T. lanuginosus* DSM 5826 (displayed on the surface of *E. coli*)*,* using thin-layer chromatography analysis (Qu et al., 2015). The content of X3 and X4 exceeded 80% of the total hydrolysis products. For EcsXyl4, the production of XOS can reach 289.5 mg/L at a substrate concentration of 1 mg/ml. In a recent study, the production of XOS reached 941 mg/L at a substrate concentration of 10 mg/ml after hydrolysis by a xylanase from *Bacillus circulans* (Kyoungseon et al., 2021). Having no xylose in an XOS mixture is an advantage because high concentrations of XOS can be obtained without a purification step (Zheng et al., 2020). Therefore, the herein-described hybrid xylanase EcsXyl4 has broad application prospects in the xylooligosaccharide industry.

### 3.6 MD simulations and protein interaction analysis

**FIGURE 8:**
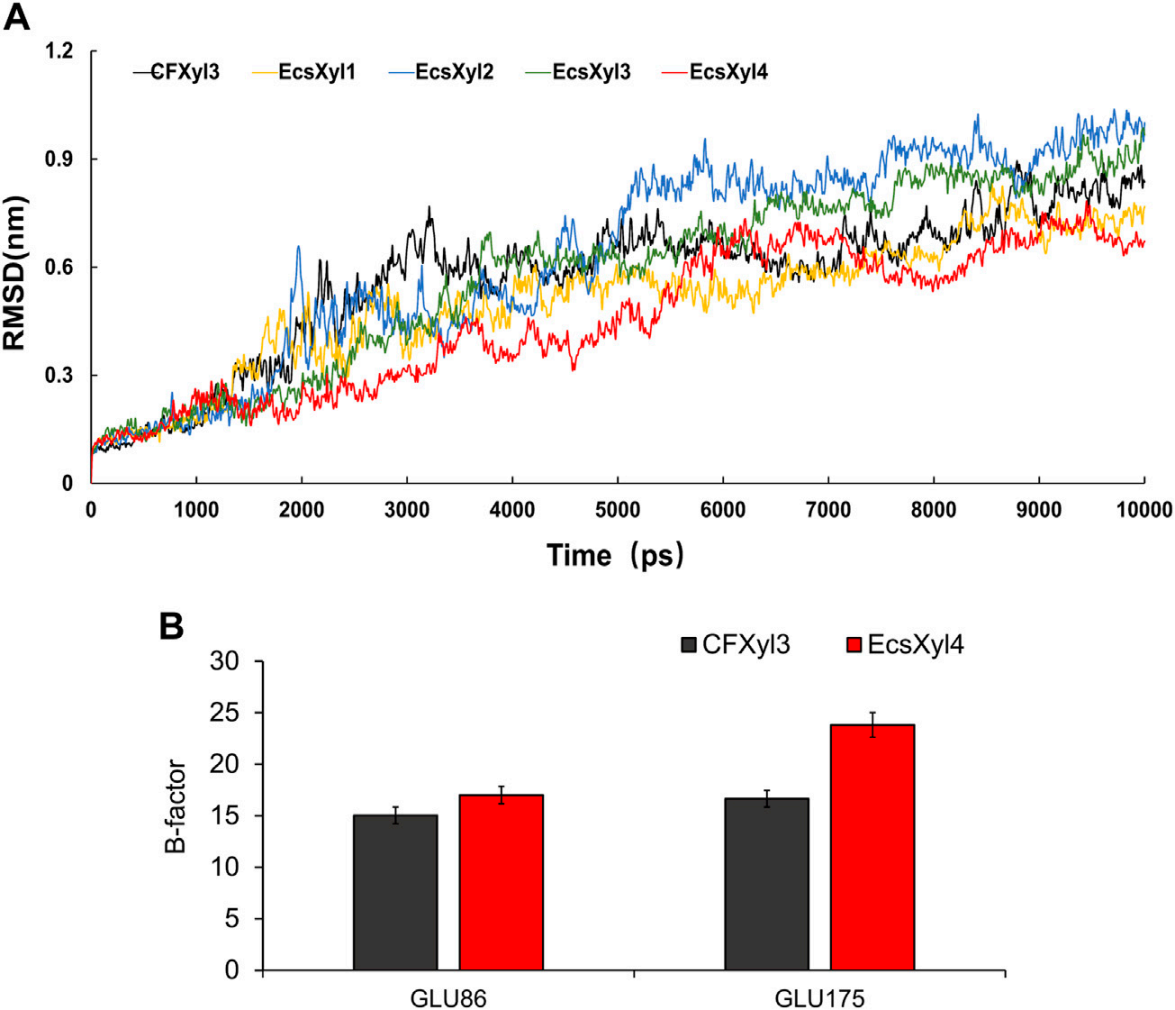
Curves of RMSD values of CFXyl3 and EcsXyl1-4 after MD simulations for 10 ns **(A)**. RMSF values of CFXyl3 and EcsXyl4 at two key catalytic sites **(B)**.

The mechanisms behind the improved thermostability of CFXyl3 and the four hybrid xylanases were analyzed using MD simulations. Overall, EcsXyl1 and EcsXyl4 displayed more stable profiles, with lower RMSD values (Figure 8A). Notably, the RMSD curves of CFXyl3 and the hybrid xylanases exhibited major fluctuations until 5,000 ps; nevertheless, the structure of EcsXyl4 became stable shortly after 6,000 ps, as depicted by the smoother RMSD curve. The low and constant RMSD of EcsXyl4 after 6,000 ps reflects the protein stability at 328 K (the optimal temperature of EcsXyl4 was 358 K). The structures of CFXyl3 and EcsXyl4 during the simulation process are shown in Supplementary Figure S3. At 300 K (before 2 ns), most of the native structure for both CFXyl3 and EcsXyl4 was maintained without any substantial changes. At 300–350 K (3–4 ns), EcsXyl4 still maintained its structure without any substantial changes, whereas the β-strands of the N-Terminus of CFXyl3 were gradually reduced. The β-strands of the N-Terminus of CFXyl3 were almost completely lost while the simulation temperature was above 400 K (5–10 ns). At 350–450 K (5–8 ns), the β-strands of the N-Terminus of EcsXyl4 reduced gradually. At 450–500 K (9–10 ns), EcsXyl4 showed a significant loss of N-Terminus β-strands. In general, the structure of EcsXyl4 was stable below 400 K, especially around the N-Terminus. Therefore, from the above results, it seems that no further usable data could be obtained if the MD simulation was continued. According to the above analysis, the RMSD value of EcsXyl4 was lower than that of CFXyl3 before 6 ns; thus, the structure was more stable.

Table 2 compares the protein interactions of four hybrid xylanases using PIC. Previous studies demonstrated that hydrophobic interactions, hydrogen bonds, and salt bridges are the dominant structural factors responsible for the thermostability of proteins (Gao et al., 2012). Table 2 shows that all the hybrid xylanases had more than the 10 hydrophobic interactions of CFxyl3, resulting in an increase in the thermal stability of the hybrid xylanases. The specific activity and T_m_ of the GH11family xylanase from *Neocallimastix patriciarum* increased 6-fold and by 12 C, respectively. This was attributed to a hydrophobic interaction between sites 50 and 201 which was introduced by directed evolution and site-directed mutagenesis (You et al., 2012). In another study, coordinating higher k-clique hydrophobic interaction clusters by site-directed mutagenesis led to the half-life and T_m_ of a xylanase from *B. circulans* increasing 78-fold and by 8.8°C, respectively. It has been demonstrated that the increased number of hydrogen bonds could enhance the protein stability (Alponti et al., 2016). Table 2 showed that the EcsXyl4 has 227 hydrogen bonds, while CFxyl3 and EcsXyl1-3 only have 224, 216, 220, and 226, respectively, which results in a more thermal stability for EcsXyl4 than for other xylanases. In a previous study, the thermal stability of a xylanase from *N. patriciarum* increased after insertion of a CBM9_1-2, which attributed to four additional hydrogen bonds (S42–S462, T59–E277, S41–K463, and S44–G371) (Miao et al., 2022). Figure 2E shows the 3D structure of EcsXyl4. Two salt bridges (Lys23–Asp24 and Glu35–Arg38) and one disulfide bridge (Cys5–Cys32) were introduced into EcsXyl2–4, resulting from N-terminus substitution, whereas only one salt bridge was introduced into EcsXyl1. The lack of the second salt bridge could explain the lower thermostability observed for EcsXyl1. A previous study showed a decrease in xylanase XynGR40 thermostability after the disruption of two salt bridges by site-directed mutagenesis (Wang et al., 2016). A more recent study showed a decrease in recombinant xylanase XynBCA thermostability after the introduction of two salt bridges (Lys187–Asp183 and Lys296–Asp300) (Mhiri et al., 2016). Aromatic interactions are thought to contribute to the stability of proteins (Perales et al., 2021). EcsXyl4 has 11 aromatic interactions (calculated by PIC), while EcsXyl1, EcsXyl2, and EcsXyl3 only have 10 each (Table 2). This resulted in the optimum temperature of EcsXyl4 being higher than that of EcsXyl1-3. A previous study (Georis et al., 2000) also confirmed that additional aromatic interactions can improved the thermostability and thermophilicity of a mesophilic family 11 xylanase.

**TABLE 2:** Comparison of protein interactions of CFxyl3 and four hybrid xylanases.

Protein interactions	CFxyl3	EcsXyl1	EcsXyl2	EcsXyl3	EcsXyl4
Hydrophobic Interactions	122	133	132	136	133
Disulphide Bridges	0	1	1	1	1
Hydrogen Bonds	224	216	220	226	227
Ionic Interactions	6	9	10	10	10
Aromatic-Aromatic Interaction	7	10	10	10	11

### 3.7 Enzymatic kinetic parameters

The kinetic parameter values of CFXyl3 and EcsXyl4 were calculated using a nonlinear regression method (Supplementary Figure S3). The K_m_ and V_max_, of CFXyl3 were 1.39 mg/ml and 1292.2 mmol/min/mg, respectively, and those of EcsXyl4 were 5.70 mg/ml and 4038 mmol/min/mg, respectively, at 55°C. Although the affinity of xylanase EcsXyl4 for the substrate was not as strong as that of CFXyl3, its catalytic ability was much higher than that of CFXyl3. The K_m_ value of EcsXyl4 for beechwood xylan was 5.70 mg/ml, which is lower than those of most reported GH family 11 xylanases from *Streptomyces* sp. J103 (51.4 mg/ml; Marasinghe et al., 2021), *Bacillus* sp. (16.4 mg/ml; Takita et al., 2021), *M. heterothallica* (13.4 mg/ml; de Amo et al., 2019), and *Fusarium* sp. (9.8 mg/ml; Li et al., 2020), but higher than that of CsXyn11A from *Chaetomium* sp. (2.84 mg/ml; Liu et al., 2021).

## 4 Conclusion

We confirmed that the thermostability of CFXyl3, a mesophilic family 11 xylanase from *C. flavigena*, was increased by replacing its N-terminal residues with the corresponding residues of SyXyn11P, a xylanase from hyperthermostable family 11. On the basis of N-terminus substitution by rational design resulting from structural analysis, EcsXyl1-4 were constructed and expressed. The apparent optimal temperature of EcsXyl4 was determined to be 85°C and its optimal pH value was 6.0. The specific activity of EcsXyl4 reached 80.90 U/mg. Our present findings make an important contribution to enhance the activity of EcsXyl1-4 in future studies. Moreover, we have engineered a xylanase (EcsXyl4) that can potentially be used for biological pulping applications.

## Data Availability

The datasets presented in this study can be found in online repositories. The names of the repository/repositories and accession number(s) can be found below: https://www.ncbi.nlm.nih.gov/, WP_013115499.1, https://www.ncbi.nlm.nih.gov/, JX459567.
