# Supplementary material for: Engineering mesophilic GH11 xylanase from Cellulomonas flavigena by rational design of N-terminus substitution
Source: Front Bioeng Biotechnol. 2022 Nov 3;10:1044291. doi: 10.3389/fbioe.2022.1044291 (PMC9669568; doi:10.3389/fbioe.2022.1044291)
Supplement: Supplementary file 1 [file DataSheet1.PDF]

Supplementary Material

Table S1. Primers used for plasmid construction in this study.

Primer Name	Primer sequence（5′-3′）
Syxyn11P-1-F	TAAGAAGGAGATATACCATGAATGCTCAGACATGTCTAACTTCACC
Syxyn11P-1-R	CTGGTGCTGTAGTTACCGCCCTCCAACAGGCAGAAATTCACG
CFXyl3-1-F	GGCGGTAACTACAGCACCAGC
CFXyl3-1-R	CATGGTATATCTCCTTCTTAAAGTTAAACA
Syxyn11P-2-F	TAAGAAGGAGATATACCATGAATGCTCAGACATGTCTAACTTCACC
Syxyn11P-2-R	AAGTTGCCCGTATTACGCCAATTGCTGGTATAACGGCCACC
CFXyl3-2-F	TGGCGTAATACGGGCAACTT
CFXyl3-2-R	CATGGTATATCTCCTTCTTAAAGTTAAACA
Syxyn11P-3-F	TAAGAAGGAGATATACCATGAATGCTCAGACATGTCTAACTTCACC
Syxyn11P-3-R	CCCTTACCAATAACAAAGTTGTTGATGCCGCTCCAATTGC
CFXyl3-3-F	AACTTTGTTATTGGTAAGGGTTGGA
CFXyl3-3-R	CATGGTATATCTCCTTCTTAAAGTTAAACA
Syxyn11P-4-F	TAAGAAGGAGATATACCATGAATGCTCAGACATGTCTAACTTCACC
Syxyn11P-4-R	CCCGTGCTCCAACCCTTACCACCAACCCAGTTGTTGATGCC
CFXyl3-4-F	GGTAAGGGTTGGAGCACGG
CFXyl3-4-R	CATGGTATATCTCCTTCTTAAAGTTAAACA

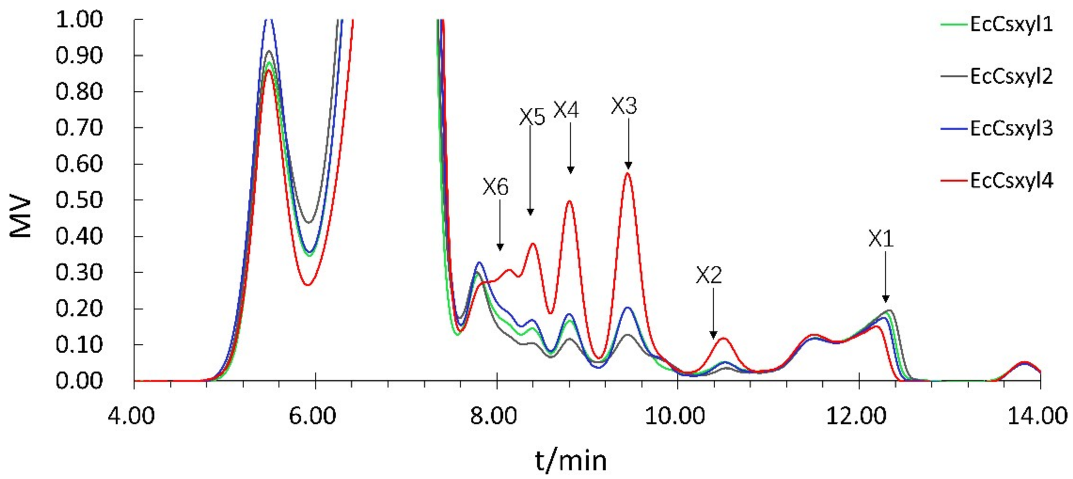

Figure S1. HPLC analysis of the hydrolystaes released from insoluble beechwood xylan by reCFxyl3 at pH 7.0 and 55°C for 15min. The positions of xylose (X1), xylobiose (X2), xylotriose (X3), xylotetraose (X4), xylopentaose (X5) and xylohexaose (X6) are shown by arrows.

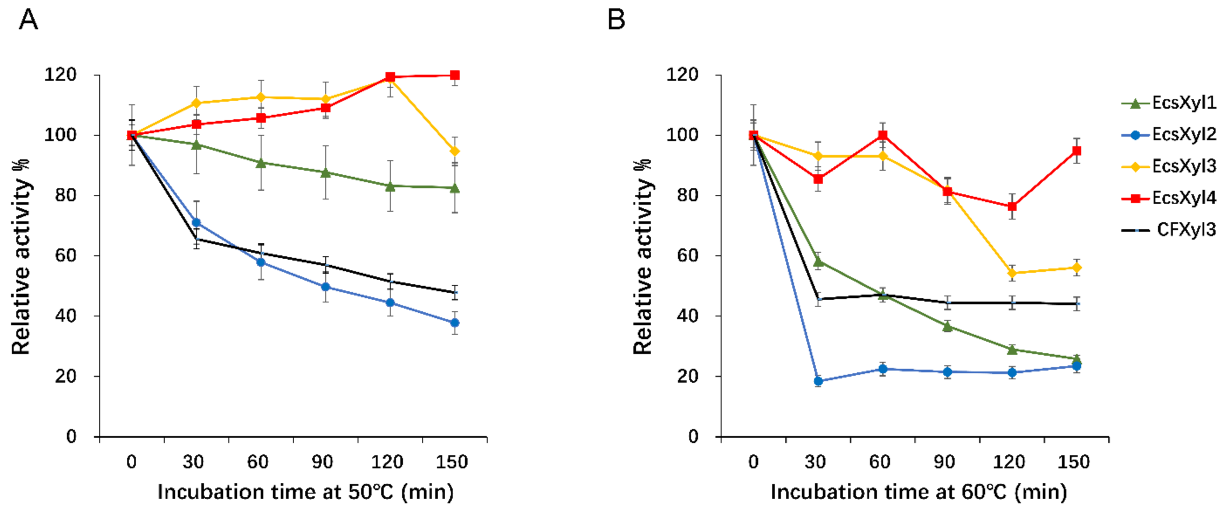

Figure S2. Thermal stability of the CFXyl3 and EcsXyl1-4 xylanases. (A): Thermal stability at 50 °C; (B): Thermal stability at 60 °C.

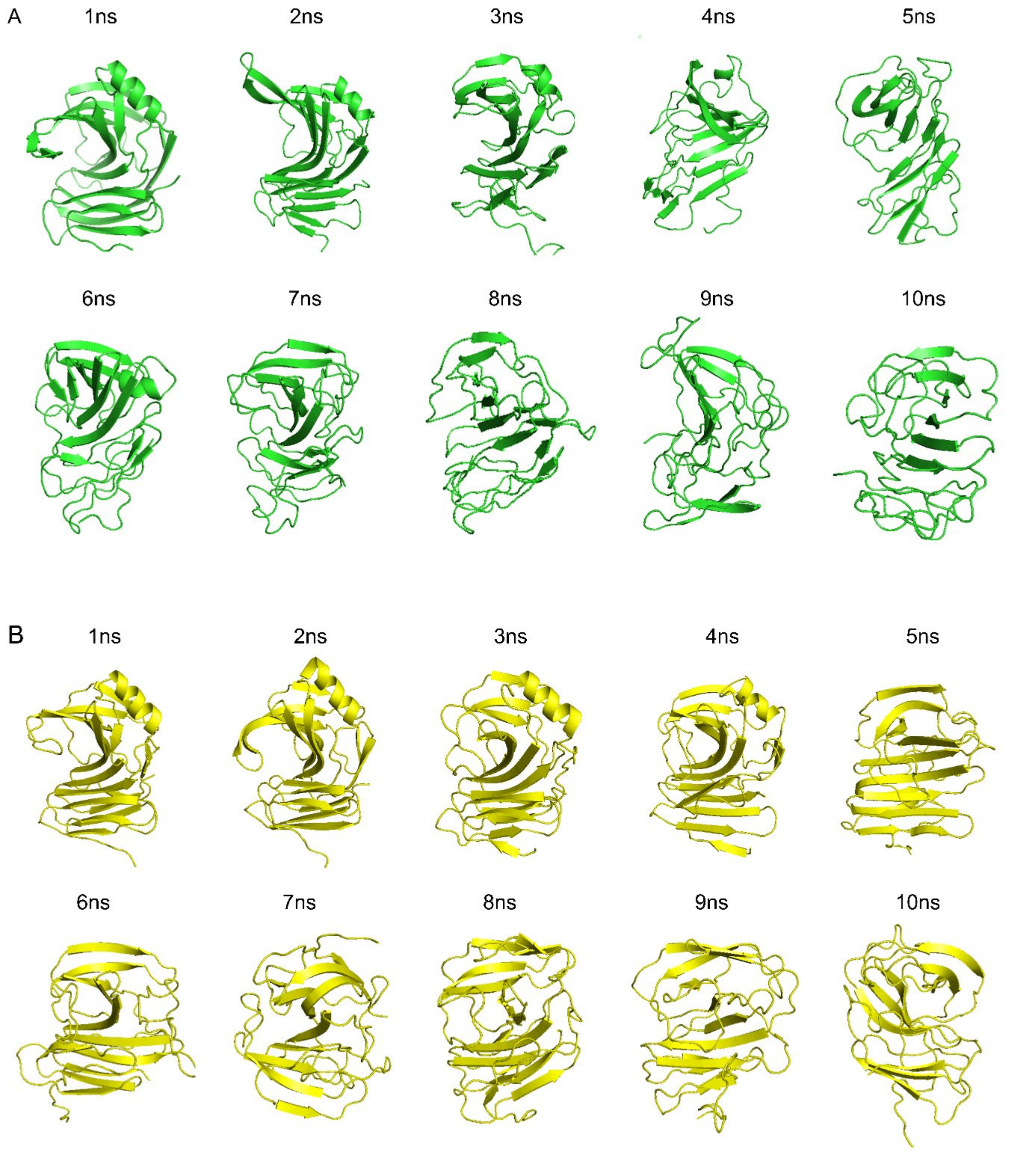

Figure S3. Molecular structure in simulation process(1-10ns). (A) CFXyl3 (B) EcsXyl4

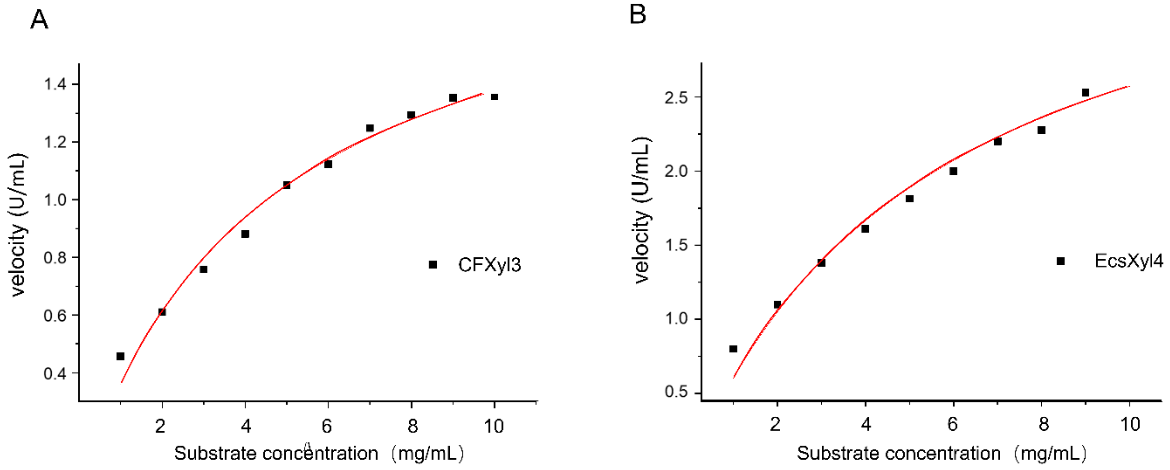

Figure S4. Nonlinear regression curves for the enzymatic reaction of xylanase. (A) CFXyl3 reaction (B) EcsXyl4 reaction
